# Facile Microwave Hydrothermal Synthesis of ZnFe_2_O_4_/rGO Nanocomposites and Their Ultra-Fast Adsorption of Methylene Blue Dye

**DOI:** 10.3390/ma14185394

**Published:** 2021-09-18

**Authors:** En-Rui Wang, Kun-Yauh Shih

**Affiliations:** Department of Applied Chemistry, National Pingtung University, Pingtung County 90003, Taiwan; transformersprime12345678@gmail.com

**Keywords:** microwave hydrothermal method, ZnFe_2_O_4_/rGO, dye adsorption

## Abstract

The industry development in the last 200 years has led to to environmental pollution. Dyes emitted by pharmaceutical and other industries are major organic pollutants. Organic dyes are a pollutant that must be removed from the environment. In this work, we adopt a facile microwave hydrothermal method to synthesize ZnFe_2_O_4_/rGO (ZFG) adsorbents and investigate the effect of synthesis temperature. The crystal structure, morphology, chemical state, and magnetic property of the nanocomposite are investigated by X-ray diffraction, Raman spectroscopy, Fourier transform infrared spectroscopy, transmission electron microscopy, and a vibrating sample magnetometer. Furthermore, the synthesized ZFGs are used to remove methylene blue (MB) dye, and the adsorption kinetics, isotherm, mechanism, and reusability of this nanomaterial are studied. The optimal ZFG nanocomposite had a dye removal percentage of almost 100%. The fitting model of adsorption kinetics followed the pseudo-second-order model. The isotherm model followed the Langmuir isotherm and the theoretical maximum adsorption capacity of optimal ZFG calculated by this model was 212.77 mg/g. The π–π stacking and electrostatic interaction resulted in a high adsorption efficiency of ZFG for MB adsorption. In addition, this nanocomposite could be separated by a magnet and maintain its dye removal percentage at almost 100% removal after eight cycles, which indicates its high suitability for utilization in water treatment.

## 1. Introduction

With global industrialization and urbanization, an increasing amount of wastewater containing a large number of organic dye molecules and heavy metals is being discharged into water systems. Consequently, global water pollution has become a serious environmental concern. Dye wastewater is mainly discharged from industries such as paper, leather, rubber, textiles, and pharmaceuticals [1]. Organic dyes do not easily decompose in the natural environment because of their stable and complex chemical structure [2]. Dye molecules may block sunlight from entering the water and reduce the dissolved oxygen level in water systems [3]. They also endanger the lives of animals and humans. For example, some organic dyes are carcinogens that inevitably have long-term effects on the human body or on unborn babies. The cationic methylene blue (MB) is a commonly used organic dye. Its chemical formula is C_16_H_18_N_3_ClS. It is used not only for dyeing paper, silk, and cotton but also for biological research [4,5]. While MB is widely used, it may cause short-term adverse effects such as breathing difficulties, vomiting, headaches, and methemoglobinemia [6]. Furthermore, MB dye has potentially life-threatening toxicity in newborns [6]. Therefore, the removal of organic dye molecules from industrial and urban effluents has become a very important issue. The dye removal process includes adsorption, coagulation, ion exchange, photocatalytic, membrane separation, and biological treatment [7,8,9]. Owing to the low cost of dye adsorption and the simplicity of design, it is the most suitable technology [10]. Therefore, many efforts have been made to develop the high adsorption performance of materials for organic dyes.

Traditionally, the most commonly used adsorbents for the adsorption of methylene blue are activated carbon and ion exchange resins. Activated carbon (AC) has been used as an adsorbent because of its large surface area, pore structure, high adsorption capacity, and high affinity for a variety of dyes. Although the effectiveness of activated carbon as an adsorbent for a wide range of pollutants is well known [11,12,13], the low adsorption capacity of a single activated carbon is sometimes not sufficient for the removal of pollutants from different types of wastewaters, and therefore the modification of activated carbon to form composite materials is increasingly being investigated. The potential of activated carbon loaded with manganese iron oxide (AC-MnFe_2_O_4_) powder is very good at removing methylene blue (MB) dye from aqueous solutions, and the maximum adsorption capacity obtained is 77.44 mg g^−1^ [14]. Ion exchange resins, on the other hand, are also commonly used as sorbents for dyes [15]. Ion exchange resins are polymers with various functional groups. These functional groups can combine with ions of opposite charge. Given that dyes are mostly positively charged, they can be removed from wastewater using anion exchange resins. Different types of ion exchange resins have been used for the adsorption of dye wastewater [16,17]. Khan et al. [18] showed the adsorption potential of Zerolit DMF (ZDMF) resin for methylene blue (MB) removal in aqueous systems. The results showed that the best adsorption (24.75 mg g^−1^) was achieved at pH 10. However, the method of their synthesis and the structure of the polymers make ion-exchange resins expensive. The manufacturing process of these materials involves suspension polymerization or solution polymerization, which generates a large percentage of chemical waste, which is a substantial environmental hazard [19]. Therefore, the development of new adsorbents has been the main direction of research in recent years.

Graphene, the two-dimensional graphite, was first isolated by A. K. Geim and K. S. Novoselov in 2004 [20]. Graphene has a higher specific surface area than many materials because it is a monolayer honeycomb lattice structure of carbon atoms with a sp^2^ orbital [21,22]. Therefore, graphene substrate as a potential dye adsorption material has received considerable attention. For example, Notley and Sham used the surfactant exfoliated graphene to adsorb MB dye [23]. Zhang et al. used photo-reduced graphene oxide (PRGO) to adsorb dye molecules and heavy metal ions [24]. Gupta et al. investigated the adsorption capacity of reduced graphene oxide (rGO) for methyl orange (MO), malachite green, and other dyes [25]. Xiao et al. combined L-cysteine with reduced graphene oxide (RGO-Cys) to remove cationic neutral red (NR) and anionic indigo carmine (IC) [26]. However, these adsorbents are difficult to separate from water, which hinders their application. To address this issue, some researchers have used graphene composites in combination with magnetic materials to remove water pollutants. Spinel ferrite, whose chemical structure is MFe_2_O_4_ (M is a divalent metal cation), is a good choice for the formation of graphene composites and dye adsorption. Given their excellent magnetic properties, nanocomposites can be magnetically separated from wastewater and then reused for the removal of organic dyes [27]. In addition, ferrites have chemical stability, good mechanical hardness, and adsorption property [28]. These reasons have made many scientists more interested in ferrite-graphene composites. For example, Li et al. synthesized poly(4-vinyl pyridine)-graphene oxide-Fe_3_O_4_ to remove MB dye from an aqueous solution [29]. Gupta and his collaborators studied the adsorption property of CoFe_2_O_4_/GO for MB and methyl violet (MV) dyes [27]. Geng et al. investigated a functional hybrid of rGO-Fe_3_O_4_ nanoparticles for dye removal [30]. Saiphaneendra and his co-workers studied the synergistic effect of rGO-Fe_2_O_3_-Fe_3_O_4_ on dye adsorption [31].

The synthesis method of the traditional hydrothermal method often heats the precursor in the vessel at high temperatures [32]. The disadvantage of this method is the large amount of energy lost in the heating process. The hydrothermal method always requires several hours, even up to a few days, to synthesize the sample. However, the heating process of the microwave hydrothermal method uses the electromagnetic wave to directly heat the samples in the vessel. This method reduces the energy lost in the heating process and decreases the time needed considerably [33]. The time cost in sample synthesis via the microwave hydrothermal method is usually less than one hour. In addition, the use of microwave preparation of samples has other advantages, fewer impurities, better reproducibility, safety, and excellent control of experimental parameters [34,35]. Undoubtedly, microwave hydrothermal is an excellent and eco-friendly method to synthesize materials.

In this study, we chose ZnFe_2_O_4_ to prepare graphene nanocomposites and use them for MB adsorption. Although the magnetization value of ZnFe_2_O_4_ is low, it is a green material with low toxicity compared to other ferrites such as CoFe_2_O_4_, NiFe_2_O_4_, and MnFe_2_O_4_ [36,37]. In addition, ZnFe_2_O_4_ also has other advantages, e.g., low eddy-current loss, high magnetic permeability, high electronic conductivity, and low synthesis cost [38]. In previous articles, zinc ferrite–graphene matrix materials were used less frequently to adsorb organic dyes. The few reports using zinc ferrite–graphene adsorbents did not show good adsorption capacity and adsorption rates. The adsorption mechanism was also not discussed in depth. Sadighian et al. used zinc ferrite-graphene for adsorption of methyl orange (MO), but the maximum adsorption capacity was only 171 mg g^−1^ and only three regeneration cycles [39]. Fei et al. used zinc ferrite-reduced graphene doped with barium (II) for adsorption of methylene blue (MB) [40]. The experimental results showed that this composite had an MB adsorption capacity of 19.03 mg g^−1^. After five cycles of testing, its capacity was only 5.87 mg g^−1^. Therefore, we believe that the true adsorption capacity of the zinc ferrite–graphene-based adsorbent has not been found yet.

The main objective of this study was to prepare zinc ferrite-reduced graphene oxide magnetic-nanoparticle (ZFG; ZF = ZnFe_2_O_4_) by the microwave hydrothermal method using a combination of low toxicity zinc ferrite and reduced graphene oxide [41]. The dye adsorption mechanism of this nano-sorbent was investigated by using ZFG to adsorb harmful MB dyes. In our work, we synthesized graphene oxide (GO) by modified Hummer’s method and ZFG nanocomposites were prepared using the microwave hydrothermal method at various temperatures. We compared the chemical state, crystal structure, surface morphology, and adsorption properties of the prepared samples to find the optimum sample parameters with the fastest absorption rate and the highest adsorption capacity. Finally, the adsorption mechanism of ZFG was investigated further using FT-IR and Raman spectrum.

## 2. Materials and Methods

### 2.1. Materials

The chemical reagents include natural graphite powder (200 mesh, UniRegion Bio-Tech, Hsinchu, Taiwan), zinc nitrate hexahydrate (99%, Alfa Aesar, Haverhill, MA, USA), ferric nitrate nonahydrate (98%, Sigma-Aldrich, St. Louis, MO, USA), sodium hydroxide (95%, Nihon Shiyaku Reagent, Kyoto, Japan), sulfuric acid (99.99%, Nihon Shiyaku Reagent, Kyoto, Japan), acetone (95%, Nihon Shiyaku Reagent, Kyoto, Japan), hydrochloric acid (37%, Union Chemical, Hsinchu, Taiwan), potassium permanganate (99.3%, Union Chemical, Hsinchu, Taiwan), sodium nitrate (99.5%, Hayashi Pure Chemical Ind, Osaka, Japan), hydrogen peroxide (30%, Showa Chemical, Tokyo, Japan), and methylene blue (99%, Koch-Light Laboratories, Ltd., Haverhill, Suffolk, UK). GO was prepared via modified Hummer’s method. The purity of the chemicals used in this experiment is of analytical grade, and they were used directly without any treatment.

### 2.2. Synthesis GO Powder

The preparation of GO was based on our previous studies [42,43] and was synthesized from natural graphite powder by a modified Hummers method using H_2_SO_4_ and KMnO_4_ as oxidants [44,45]. The 69 mL pure sulfuric acid solution was taken into the flat bottom flask and given an ice bath to 5 °C. Then, graphite powder, NaNO_3_, and KMnO_4_ were added into the flask and stirred in the ice bath for 1.5 h. The mixture temperature was maintained at 40 °C 30 min. Next, 120 mL of DI-water was added into the flask and stirred for 30 min. Added 300 mL DI-water and 9 mL of 30% hydrogen peroxide were added to stop the reaction. The product was filtered and placed in 500 mL of 1.6 M hydrochloric acid and stirred 30 min for removing the remaining metal ions. The prepared GO was washed with deionized water to remove residual ions and acids [46]. Finally, the GO suspension was filtered and dried in a 70 °C oven.

### 2.3. Synthesis of ZFG Nanoadsorbent

**Figure 1:**
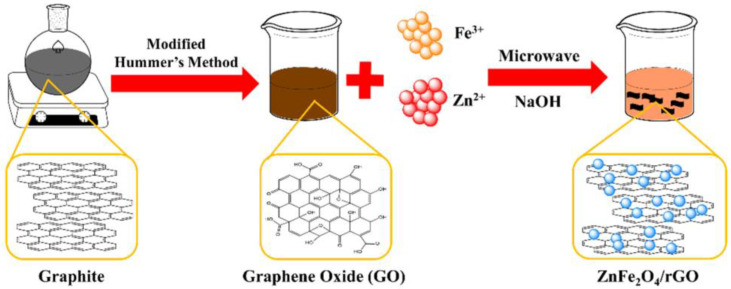
Scheme of the preparation of ZFG nanomaterial.

For synthesizing the ZFG nanoadsorbents, 50 mg of GO was dispersed in 30 mL deionized water ultrasonically at room temperature. After adding 0.42 mmol Fe(NO_3_)_3_ and 0.21 mmol Zn(NO_3_)_2_, this solution was treated ultrasonically for 30 min, using 2M NaOH to adjust the solution pH value to 12. After ultrasonication for 15 min, the solution was moved to a Teflon-lined vessel and using a microwave heating sample. We controlled the heating temperature at 140, 160, 180, and 200 °C to synthesize various materials of ZFG (ZFG-14, ZFG-16, ZFG-18, and ZFG-20). Finally, the ZFGs were filtrated and dried in an oven at 75 °C for 12 h. Pure ZF and rGO were synthesized at 180 °C by a similar process. Figure 1 illustrates the scheme of the preparation of ZFG nanomaterial.

### 2.4. Characterizations

ZFG nanomaterials were prepared using the Flexiwave T660 (Milestone srl, Sorisole, Italy). A crystal of the as-prepared samples was examined using X-ray diffraction measurements (D8 Advance diffractometer, BRUKER Co. Ltd., Billerica, MA, USA). The X-ray diffractometer used Cu Kα radiation (λ = 1.5418 Å). The measurement voltage and current are 40 kV and 25 mA, respectively. The morphology of the particles was characterized by transmission electron microscopy (TEM, H7500, Hitachi High-Tech. Co. Ltd., Tokyo, Japan), the accelerating voltage was 80 kV, and the magnification was 200 kx. Raman spectra were obtained with a nitrogen-cooled CCD detector (Shamrock 750 spectrograph, Andor Technology Co. Ltd., Belfast, UK). A randomly polarized 533 nm laser was used to excite at 0.45 mW laser power. The magnetic properties at room temperature were analyzed by a vibrating sample magnetometer (VSM, 7400 Series, Lake Shore Cryotronics, Westerville, OH, USA) in the applied field of H = ±15 kOe. The functional group of nano-adsorbents used a Fourier transform infrared spectrometer (FT/IR-6700 spectrometer) from JASCO International Co., Ltd., Tokyo, Japan. The nitrogen adsorption–desorption isotherm was measured by the Micro 100C (3P Instruments Co. Ltd., Odelzhausen, Germany) under 77 K. The prepared sample was degassed under vacuum at 120 °C for 12 h before measurement. The concentration of dyes solution was determined with CT-2200 UV/Vis spectrophotometer delivered by ChromTech Co. Ltd., Apple Valley, MN, USA.

### 2.5. Dye Adsorption

The adsorption ability of ZFGs was evaluated by studying its adsorption of MB. In this study, 10 mg as-synthesized samples were added to a 10 mg L^−1^ 50 mL MB aqueous solution. The solution was magnetically stirred and the temperature was controlled at 295 K. At each predetermined time (3 min), 9 mL dye solution was drawn out and centrifuged. After centrifugation, the concentration of MB solution was determined by a UV-visible spectrophotometer at 664 nm. At the end of the experiment, the Dye removal percentage and adsorption capacity ($q_{t}$) were calculated using Equations (1) and (2) [47].

(1) $$Dye\;removal\;\left(\%\right)=\left(\frac{C_{0}-C_{t}}{C_{0}}\right)\times 100\%,$$

(2) $$q_{t}=\frac{(C_{0}-C_{t})\times V}{M}.\;$$

Here, $C_{0}$ is the initial concentration of the solution (mg L^−1^); $C_{t}$ is the concentration at time *t*; V is the volume of the dye solution (L), and M is the mass of adsorbent (g) [47,48]. When the adsorption process achieves equilibrium, the equilibrium adsorption capacity ($q_{e}$) can be calculated by the following equation:

(3) $$q_{e}=\frac{(C_{0}-C_{e})\times V}{M},$$

where $C_{e}$ is the equilibrium concentration of MB solution [32].

The adsorption isotherm experiments were carried out using 10 mg of adsorbent and 10–40 mg L^−1^ dye solution (50 mL). The adsorbent and dye solution were mixed and adsorption equilibrium was reached under magnetic stirring for 30 min. Equation (3) was used to investigate the experimental results by fitting Langmuir, Freundlich, and Temkin models.

The effect of pH on the adsorption was investigated using 10 mg of adsorbent and 10 mg L^−1^ MB solution. The pH range of dye solution was controlled at 1.0 to 13.0 by 0.01 M HCl and NaOH solution. This solution was magnetically stirred for 30 min at 295 K. To test the recycling performance, the ZFG was separated from the solution with a magnet after the first adsorption. This nano-adsorbent was washed with ethanol and DI water several times. After drying in an oven at 75 °C for 12 h, a fresh dye solution (10 mg L^−1^) was added for the next stage of the test, in which the efficiency of the recycled ZFG would be determined.

## 3. Results and Discussion

### 3.1. Structural Characterization

#### 3.1.1. XRD Analysis

**Figure 2:**
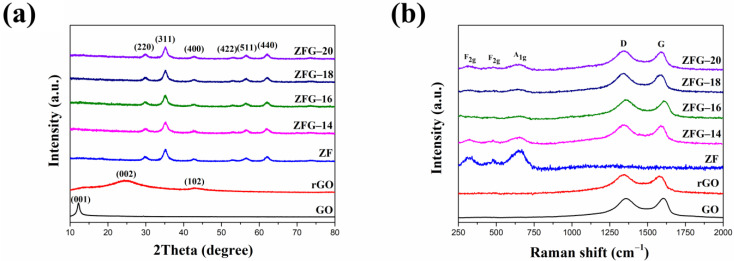
(**a**) XRD pattern and (**b**) Raman spectra of GO, rGO, ZF, and ZFGs.

The crystal structure of the ZFG nanocomposites was characterized by XRD. The measurement results are shown in Figure 2a. The XRD pattern of GO has a strong and sharp peak at 2θ = 12.2°, which corresponds to the (001) crystal plane [49]. For rGO, the disappearance of the diffraction peak on the GO (001) crystal plane indicates the complete reduction of GO by the microwave hydrothermal method. The X-ray diffraction results of rGO show two broad diffraction peaks at 2θ = 24.4° and 42.7° are the (002) and (102) planes of the rGO structure, respectively. [50]. The nanocomposites synthesized at different temperatures and pure ZF have six distinct peaks at 2θ = 29.92°, 35.27°, 42.92°, 53.11°, 56.69°, and 62.16°, which belong to spinel cubic structure ZnFe_2_O_4_ (JCPDS No. 22-1020, space group: Fd3m) [51,52]. The absence of any peak from Fe_2_O_3_, ZnO, and other crystal phases implies that the crystal phase is pure zinc ferrite. It also verifies that these nanocomposites were prepared successfully. The crystal plane of graphene oxide (001) disappears in each ZFG, which proves that the graphene oxide was successfully reduced. In addition, the rGO diffraction peaks did not appear in the XRD pattern of all ZFGs because the anchoring of ZF nanoparticles prevented the restacking of graphene nanosheets.

#### 3.1.2. Raman Spectrum

The Raman spectra of ZF, GO, rGO, and ZFG at various synthesis temperatures are shown in Figure 2b. Spinel-type ZF and ZFGs have shown similar features in the low-frequency area (100~1000 cm^−1^), which corresponds to the A_1g_ symmetry and F_2g_ asymmetric mode of spinel-type zinc ferrite (Figure S1). Wave numbers at 644 cm^−1^ are A_1g_ mode, representing the symmetric stretching of oxygen atoms in tetrahedral AO_4_ groups [53]. Wave numbers 314 and 478 cm^−1^ are F_2g_ phonon modes, which cause the asymmetric stretching and bending of oxygen in the octahedral groups (BO_6_) [53,54]. GO, rGO and ZFGs have two obvious peaks nearby at 1341 and 1589 cm^−1^, assigned to the D band and the G band of Raman spectrum of graphene, respectively. The G peak corresponding to an E_2g_ phonon mode of graphite belongs to the vibration of the sp^2^ -bonded carbon atoms [55,56]. The D peak corresponds to a k-point phonon mode of A_1g_ symmetry that comes from the sp^3^ defects within the carbon, such as vacancies, and edge effect [57]. According to the D peak and the G peak appearance, the existence of rGO in all ZFGs was confirmed. In addition, data presented in Table 1 indicates that the D and G peaks of rGO and ZFG have a red shift compared to GO, which is attributed to the reduction of GO by the microwave hydrothermal method. The ZF nanoparticles anchoring on the rGO surface caused a local compressive strain on the rGO surface [58]. This lets the G band of ZFGs have a blue shift than rGO [58]. The ratio of D-band to G-band intensity (I_D_/I_G_) of rGO and all ZFGs is higher than the GO I_D_/I_G_ value. This means rGO and ZFGs have more surface defects. However, the I_D_/I_G_ value of each ZFG has not significantly changed, and the degree of defect of each ZFG is similar.

#### 3.1.3. FTIR Spectrum

**Figure 3:**
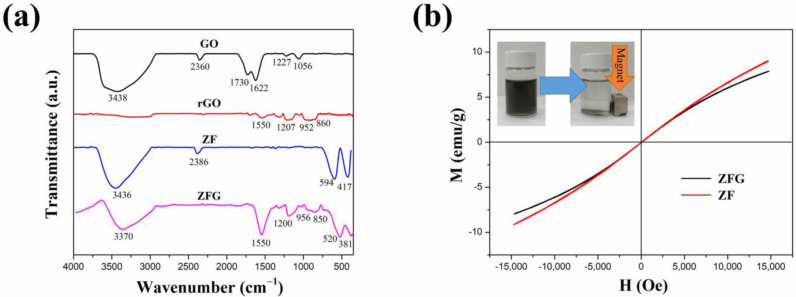
(**a**) FT-IR spectra of GO, rGO, ZF, and ZFG and (**b**) magnetization hysteresis loops of ZFG and ZF.

Figure 3a shows the FT-IR spectrum of GO, rGO, ZF, and ZFG. In the FT-IR spectra of GO, there are obvious peaks at 1056, 1227, 1622, 1730, 2360, and between 3000 and 3700 cm^−1^. The peaks at 1056 and 1227 cm^−1^ show the C-O stretching vibrations of epoxy groups and alkoxy groups [59]. The peak at 1730 cm^−1^ shows the vibration modes of the C=O group [60]. The C=C skeletal stretching vibration of GO can be seen at 1622 cm^−1^ [61]. As usual, the absorption band of GO at the range from 3000 to 3700 cm^−1^ is attributed to the bending vibration of O-H of adsorbed water and hydroxyl groups [61]. The absorption peak of CO_2_ is at 2360 cm^−1^ [62]. As per the result, GO nanosheets have abundant oxygen functional groups at the edges and basal plane. For rGO and ZFG FT-IR spectra, the absorption band located at 1550 cm^−1^ is a C=C skeletal stretching vibration of graphene structure [63]. The absorption band at 850 to 956 cm^−1^ is the overlapped region of epoxide, hydroxyl, C=O contribution, and carboxyl group [64]. The absorption bands of ZFG at 1200 and 3370 cm^−1^ are attributed to the residual C-O group and the adsorbed water [59]. The intensity of absorption bands of oxygen functional groups in ZFG and rGO decrease dramatically or even disappear, which proves that GO had been successfully reduced by the microwave hydrothermal method. The high-intensity peaks of ZF samples at 3436 and 2386 cm^−1^ are the O-H banding of water molecules adsorbed on the surface of nanoparticles and carbon dioxide adsorption in air, respectively [62]. The measurements of ZF and ZFG nanoparticles showed two distinct peaks at 520 cm^−1^ and 381 cm^−1^. These two peaks correspond to the stretching vibration of the Zn-O bond at the tetrahedral position and the Fe-O bond at the octahedral position, respectively [63,65].

#### 3.1.4. Magnetic Property

Figure 3b shows the vibrating sample magnetometer (VSM) results of ZF and ZFG at room temperature. It is found that the curves of all samples pass through the origin, which means that the material does not have the coercive force ($H_{C}$) and residual magnetization ($M_{r}$), associated with superparamagnetic nanomaterials [66]. The values of the saturation magnetization of ZFG and ZF are 14.1 emu g^−1^ and 11.7 emu g^−1^, respectively. The magnetic properties of the ZFG are lower than ZF because of the addition of non-magnetic rGO [67]. This nanomaterial can be magnetically isolated from the wastewater using a magnet (inset of Figure 3b). Therefore, the nanocomposite material is a recyclable adsorbent that can be applied in the removal of organic wastewater dyes.

#### 3.1.5. Morphological Characterization

**Figure 4:**
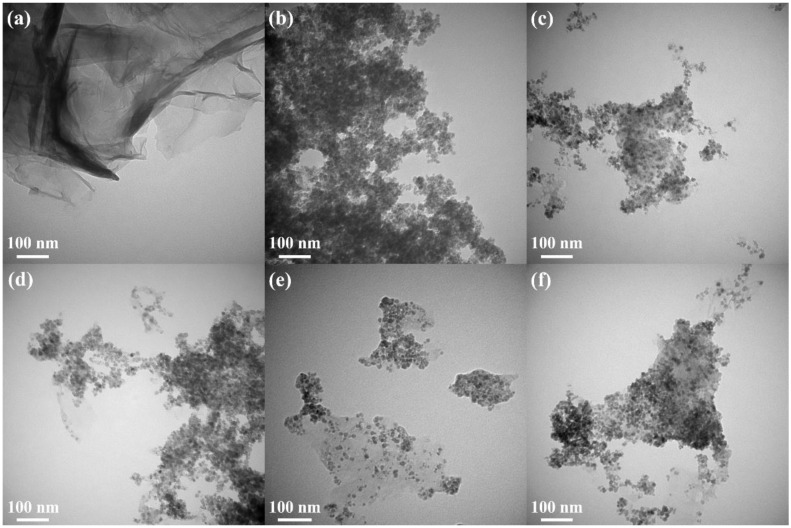
TEM image of (**a**) rGO, (**b**) ZF, (**c**) ZFG-14, (**d**) ZFG-16, (**e**) ZFG-18, and (**f**) ZFG-20.

Transmission electron microscopy (TEM) is an important and frequently used technique for the characterization of nanomaterials. The morphology of ZFG nanocomposites and the particle sizes of various synthesized samples can be observed in the TEM image. Figure S2 is the TEM image of GO, which shows a typical 2D sheet almost as transparent as a thin film and consists of wrinkles and folds that can be used to anchor ZF particles [68]. The TEM image of rGO (Figure 4a) also shows the typical 2D sheet morphology with a large number of wrinkles and folds. Figure 4b is the TEM image of pristine ZF nanoparticles, prepared without the addition of GO. The nanoparticles are nearly cubical in shape, with an average size of 12.1 nm and tending to aggregate randomly [63]. Figure 4c–f shows TEM images of ZFG nanocomposites at various synthesis temperatures. A relatively uniform decoration of ZF nanoparticles on the surface of reduced graphene oxide can be observed. ZFG-18 has a more uniform ZF nanoparticle distribution compared to other ZFGs. It can be shown from the histogram of the particle size distribution (Figure S3) and Table 2 that the average particle sizes of ZFG-14, ZFG-16, ZFG-18, and ZFG-20 are 10.6, 10.0, 8.1, and 9.3 nm, which were obtained by random measurements. The particle size decreases when the temperature increases to 180 °C, owing to the larger number of seeds growing and the more thorough particle reaction [69]. Under this optimal temperature condition, the nucleation time is short and nucleation is fast [70,71]. Therefore, ZnFe_2_O_4_ nanoparticles at 180 °C have the smallest particle size and narrower distribution width (8.1 ± 1.8) consistent with the result that short nucleation and crystallization time can produce small particle size and high dispersion [72]. The average particle size of all ZFGs is smaller than pure ZF, which confirms that the presence of rGO can effectively prevent ZF nanoparticles from aggregating with each other. Furthermore, the ZF nanoparticles can prevent the overlap of the reduced graphene oxide [73].

#### 3.1.6. N_2_ Adsorption–Desorption Measurement

**Figure 5:**
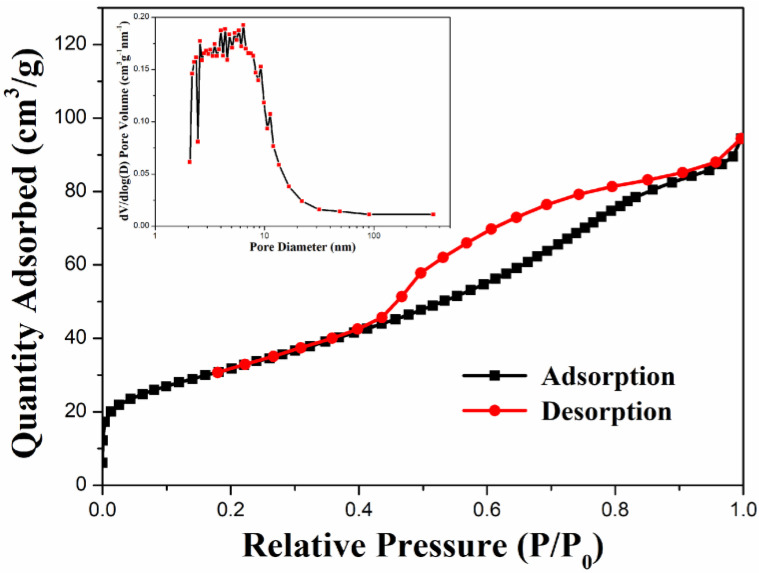
N_2_ adsorption–desorption isotherm of ZFG and pore size distributions calculated from the BJH model.

The specific surface area and porosity distribution of the nanocomposite can be confirmed by nitrogen adsorption–desorption measurements. Figure 5 shows the N_2_ adsorption–desorption isotherm of ZFG. According to the measurement results, ZFG presents type IV isotherms [74]. The adsorption capacity of N_2_ increases gradually and does not show limiting adsorption at high relative pressure (P/P_0_). There is an obvious hysteresis loop when P/P_0_ ranges from 0.44 to 0.91 due to multilayer physisorption and capillary condensation [75]. These phenomena indicate that the N_2_ adsorption isotherm of ZFG has an H4 type of hysteresis loop. The Brunauer, Emmett, and Teller (BET) surface area of ZFG is 114.30 m^2^ g^−1^. The insert figure shows the porosity distribution of ZFG, calculated by the Barrett, Joyner, and Halenda (BJH) method [76]. The porosity sizes range from 2.1 to 351.1 nm. The most frequent pore size, average pore size, and total pore volume are 2.3 nm, 5.1 nm, and 0.15 cm^3^ g*^−^*^1^, respectively.

### 3.2. Dye Adsorption Study

#### 3.2.1. Effect of Contact Time

**Figure 6:**
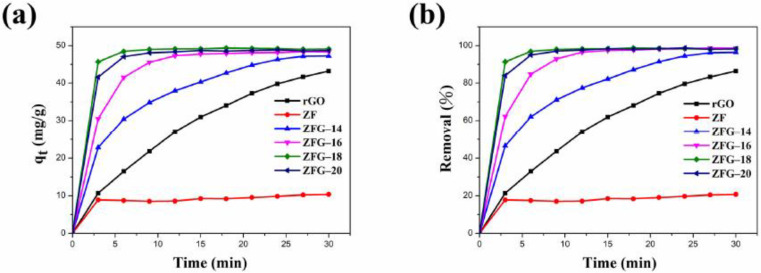
ZF, rGO, and ZFGs (**a**) adsorption capacity and (**b**) dye removal percentage. Conditions: adsorbent dosage = 10 mg, MB concentration = 10 mg L^−1^, T = 295 K.

Figure 6a,b show the adsorption capacity and dye removal efficiency of ZF, rGO, and various ZFG nanomaterials on MB solutions. In the first 3 min, the adsorption capacity and removal efficiency of the dye increased rapidly with time. After 3 min, the adsorption efficiency of these materials decreased significantly. This may be attributed to the adsorbents having many vacant surface sites during the initial stage of adsorption [77]. After a period of time, the number of these sites is occupied by dye molecules and decreases, resulting in repulsive forces on the surface of the adsorbent [77]. Consequently, the adsorbent does not readily adhere to the surface of the material. As revealed in Table 3, the equilibrium adsorption capacity (q_e_) of ZFG-18 is 49.05 mg g^−1^, higher than rGO, ZF, and other ZFGs. Furthermore, owing to the synergistic effect of rGO and ZF, the removal percentage of ZFGs is more than 96%.

#### 3.2.2. Adsorption Kinetic Study

In addition to the adsorption capacity, the adsorption kinetic constant is also an important parameter to investigate the adsorption process. The smaller kinetic constants dramatically increase the time needed to remove pollutants from the solution, owing to inefficient adsorption processes. In this study, the experimental data were examined using the pseudo-first-order, pseudo-second-order, and Elovich kinetic models. The linearized pseudo-first-order kinetic model is used widely and shown in the following equation:

(4) $$\log\left(q_{e}-q_{t}\right)=\log q_{e}-\frac{k_{1}t}{2.303},$$

where $q_{t}$ (mg g^−1^) is the absorption capacity at a certain time t (min), $q_{e}$ (mg g^−1^) is the capacity at equilibrium, and $k_{1}$ (min^−1^) is the adsorption rate constant [78]. The Lagergren pseudo-second-order model of dye adsorption is given as follows:

(5) $$\frac{t}{q_{t}}=\frac{1}{k_{2}q_{e}{}^{2}}+\frac{t}{q_{e}},$$

where $k_{2}$ (g mg^−1^ min^−1^) is the adsorption rate constant [78,79]. The linear form of Elovich model equation can be expressed as:

(6) $$q_{t}=\frac{1}{\beta }\ln\left(\alpha \beta \right)+\frac{1}{\beta }\ln\left(t\right),$$

where β is the extent of surface coverage (g mg^−1^), and α is the initial sorption rate (mg g^−1^ min^−1^) [79].

**Figure 7:**
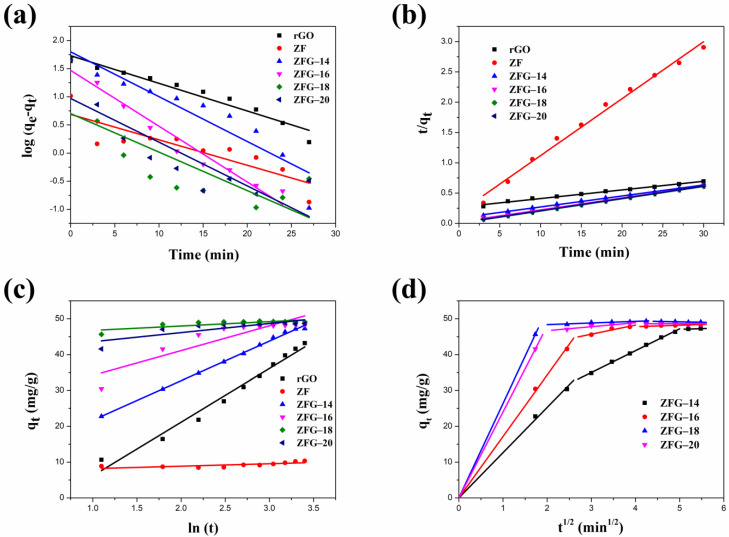
Different kinetic fitting models of MB dye adsorption (**a**) pseudo-first-order (**b**) pseudo-second-order (**c**) Elovich; (**d**) intra-particle diffusion model of ZFGs. Conditions: adsorbent dosage = 10 mg, MB concentration = 10 mg L^−1^, T = 295 K.

The linear fitting plots of these kinetic models are shown in Figure 7a–c. The fitted parameters of the pseudo-first-order, pseudo-second-order, and Elovich kinetic models are presented in Table 4. Owing to the highest correlation coefficient (R^2^) of the experimental data fitting (R^2^ > 0.99), the pseudo-second-order model is more suitable than the pseudo-first-order model and the Elovich kinetic model [80]. Furthermore, we can observe that the pseudo-second order model’s rate constant values of rGO were smaller than ZF and ZFGs, indicating that the adsorption rate of rGO is the slowest. Although ZF has a rapid adsorption rate it has the lowest adsorption capacity when adsorption reaches equilibrium. The order of the values of the kinetic constants for various ZFGs is ZF-18 > ZF-20 > ZF-16 > ZF-14. The results show that the nanocomposite synthesized at 180 °C has the fastest adsorption rate. The ZFG-18 has a more homogeneous distribution of ZF nanoparticles, as the TEM image shows. It has been reported that the dye adsorption rate of small particle material was higher than that of larger particle material [81]. Although the differences in particle sizes of ZFG-14, ZFG-16, ZFG-18, and ZFG-20 are not significant, the uniformity of nanoparticle distribution also affects the adsorption activity. The adsorption of MB dyes on ZFG nanomaterials must have stronger intermolecular interactions than the adsorption of inert gases, where the adsorption of MB requires electrostatic and π–π interactions [82]. The adsorption activity must be influenced by the diffusion of MB molecules and the interaction between ZFG and MB [83,84]. If nanoparticles can be evenly distributed on graphene, not only will the surface area increase but the active sites will also be evenly distributed. The more uniform the distribution of active sites, the more easily MB molecules diffuse to the active sites. The longer the distance between two activation sites, the less interaction between them, and the easier it is for MB to adsorb on the surface. In addition, according to the hypothesis of Langmuir isotherm, one activation point can only adsorb one molecule [85]. If two activation sites are too close together when interacting with MB molecules, a steric effect may occur, reducing the interaction between MB and ZFG. From the TEM images of ZFG, we can see that the ZFG-18 image has a more uniform distribution of ZF particles, indicating that it has the highest adsorption ability. The intra-particle diffusion model described the adsorption process of a porous adsorbent. The uptake of the adsorbate is related to the square root of time:

(7) $$q_{t}=K_{d}t^{1/2}+C,$$

where $K_{d}$ (mg min^1/2^ g^−1^) is the intra-particle diffusion rate and C (mg g^−1^) is a constant [86]. Figure 7d shows the intra-particle diffusion model of all ZFG nanocomposites. The figure shows that all ZFGs have three linear stages, which correspond to different stages in adsorption. The first stage indicates that the MB molecules pass through the boundary between the adsorbent and the liquid, adsorb on the outer surface of the material, and diffuse in the larger area of the pore [87]. In this stage, the higher concentration of MB in the solution has a stronger driving force, allowing MB to diffuse rapidly to the ZFG surface. In the second stage, after the outer surface was saturated, the adsorbate diffuses into a smaller area of the pore [87]. At this time, the diffusion rate decreases, so the adsorption rate at this stage decreases significantly. In the final stage, the diffusion rate remains constant at adsorption equilibrium. Therefore, we can know that the adsorption of ZFGs relates to the diffusion of dye molecules into the pores of ZFG nanocomposites and the adsorption on the available surface [87,88].

#### 3.2.3. Adsorption Isotherm Study

The adsorption isotherm models can be used to obtain the adsorption behavior of adsorbents. In this study, the adsorption behavior of ZFG was investigated using three isotherm models (Langmuir, Freundlich, and Temkin). The Langmuir model was proposed in 1918 by Irving Langmuir [89]. It was primarily designed to describe gas-solid phase adsorption. This model is based on the following four assumptions: (1) the adsorption process takes place at a specific and identical activity site within the adsorbent; (2) each adsorbate molecule occupies one activity site; (3) the adsorbate molecules adsorbed on the adsorbent surface without any interaction to alter adsorption behavior; (4) the monolayer adsorbed molecules covered the adsorbent surface [90,91]. The linear form of the Langmuir isotherm is given by Equation (8):

(8) $$\frac{C_{e}}{q_{e}}=\frac{C_{e}}{q_{m}}+\frac{1}{q_{m}K_{L}},$$

where $C_{e}$ is the equilibrium concentration (mg L^−1^), $q_{m}$ is the maximum dye adsorption capacity (mg g^−1^), $q_{e}$ is the amount of dye adsorbed per gram adsorbent at the adsorption equilibrium, and *K_L_* is the Langmuir constant (L mg^−1^) [92,93]. In addition, a dimensionless constant called separation factor (*R_L_*) can evaluate the favorability of the adsorption process (as given in Equation (9)):

(9) $$R_{L}=\frac{1}{1+C_{0}K_{L}},$$

where *R_L_* > 1.0 represents an unfavorable adsorption process; *R_L_* = 1.0 is a linear adsorption process; 0 < *R_L_* < 1.0 denotes favorable adsorption, and *R_L_* = 0 is irreversible adsorption [94]. The Freundlich isotherm was derived as an empirical relationship in the sorption process at first. This isotherm describes the surface heterogeneity adsorption process, and the exponential distribution of adsorption energies and active site distribution [91]. Therefore, this model is mainly applied with multi-layer and heterogeneous surface adsorption. The mathematical model can be written as the following equation:

(10) $$lnq_{e}=lnK_{f}+\frac{1}{n}lnC_{e},$$

where, $K_{f}$ (L mg^−1^) and n are the Freundlich constant and the intensity of adsorptive bond and bond distribution, respectively [95]. The 1/n value is a useful parameter because it can estimate the surface heterogeneity of the adsorbent [91]. When this value is close to zero, the surface of the adsorbent becomes more heterogeneous. Moreover, the n value varies with the heterogeneity of the adsorbent; 0 < 1/n < 1 indicates that the adsorption process is favorable [96]. The Temkin model assumes that the heat of adsorption (ΔH_ads_) of molecules within the layer decreases linearly and also considers the effect of adsorbate/adsorbate interactions on the adsorption process [97,98]. The Temkin isotherm has the linear form presented in Equation (11):

(11) $$q_{e}=BlnA_{T}+BlnC_{e}.$$

In this equation, parameter $A_{T}$ is the Temkin isotherm constant (L g^−1^) and constant B is the Temkin isotherm energy related to the heat of adsorption (kJ mol^−1^) [99].

**Figure 8:**
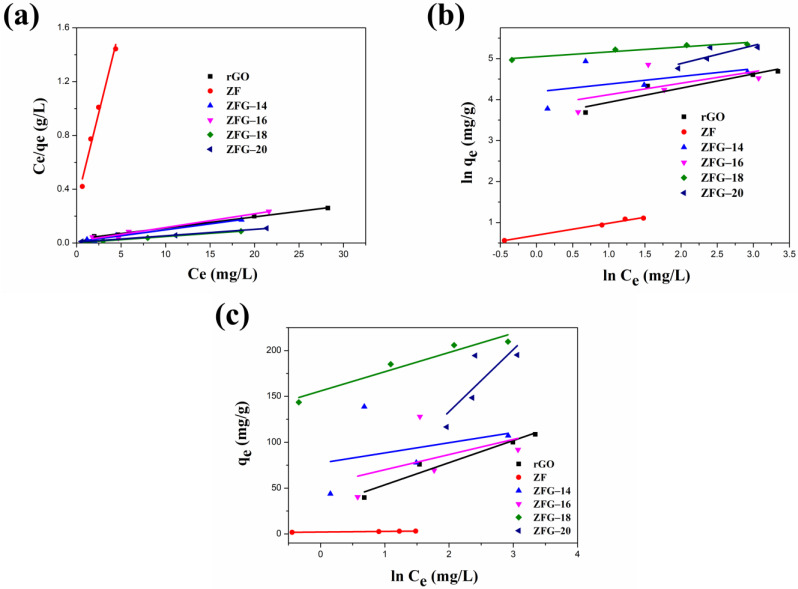
Linear fitting of (**a**) Langmuir, (**b**) Freundlich, and (**c**) Temkin adsorption isotherm. Conditions: adsorbent dosage = 10 mg, time = 30 min, MB concentration = 10 mg L^−1^, T = 295 K.

Figure 8 and Table 5 present the fitting plots and calculated parameters of Langmuir, Freundlich, and Temkin isotherms for the various materials. Similar to the adsorption kinetics discussed earlier, the correlation coefficient (R^2^) is an important parameter in determining the models that are optimal for describing the ZFG adsorption process [100,101]. The R^2^ values (R^2^ > 0.96) of the Langmuir model for all various samples were higher than Freundlich and Temkin isotherms. This result illustrates that the adsorption phenomenon is a monolayer on the energetically equivalent and homogeneous distribution activity sites of ZF, rGO, and each ZFG nanoadsorbent. The *R_L_* values for all materials in the range of 0 < *R_L_* <1 indicate beneficial adsorption to MB. Furthermore, the theoretical maximum adsorption capacity of ZFG-18, calculated using the Langmuir model is 212.77 mg g^−1^ higher than ZF, rGO, and other ZFGs. To compare the adsorption capacity of MB in various studies, Table 6 presents the maximum adsorption capacity of ZFG-18 and some previous reports. From this table, it can be observed that ZFG-18 nanomaterials exhibit excellent adsorption performance.

#### 3.2.4. Effect of pH Values

**Figure 9:**
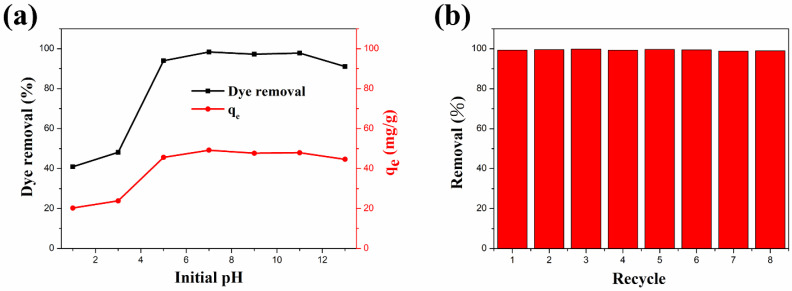
(**a**) Adsorption behavior of ZFG-18 affected by the dye solution initial pH. (**b**) Dye adsorption recycle test of ZFG-18. Conditions: adsorbent dosage = 10 mg, time = 30 min, MB concentration = 10 mg L^−1^, T = 295 K.

The pH of the dye solution can significantly affect the intermolecular interactions between adsorbents and dye molecules [110]. Therefore, pH is an extremely significant parameter in dye adsorption studies. The influence of the ZFG-18 dye adsorption capacity by solution initial pH is illustrated in Figure 9a. In this study, 10 mg of adsorbent was added to 50 mL of 10 mg L^−1^ MB dye solution with a pH range of 1–13. According to the experiment result, the removal percentage and adsorption capacity increased with the increase of the initial pH. The reason for this result is possibly that the initial pH of the solution changes the charge states on the adsorbent surface. In acidic solutions, ZFG has a more positive surface charge because many H^+^ ions in the dye solution interact with the residual oxygen-containing groups on the nanocomposite surface [29]. This leads to the adsorbent surface being more positively charged and decreases the removal percentage of the cationic dye methylene blue. Furthermore, the alkaline solution causes deprotonation of residual oxygen-containing groups on the surface and edges of the rGO. The deprotonation causes a more negative charge at the adsorbent surface and improves the adsorption ability of MB molecules. Therefore, electrostatic attraction is important in the process of organic dyes adsorption by ZFG [29].

#### 3.2.5. Recycle Test

In addition to the high dye molecules removal ability and removal rate, the recycle life of adsorbents is also important. The recyclability study of the ZFG-18 nano-adsorbent was carried out with an adsorption recycle test. The recycle test also uses the 10 mg L^−1^ MB solution. After each use, the ZFG was recycled as follows: it was isolated using the magnet, washed with alcohol and DI-water separately, and dried in the oven for the next cycle. The experiment results are shown in Figure 9b. The first cycle of this adsorbent removes almost 99.29% dye molecules from the solution. In addition, the MB dye removal was more than 99% after eight cycles. This result illustrates that ZFG-18 has a long recycle life and adsorption ability and could be reused.

#### 3.2.6. Adsorption Mechanism

**Figure 10:**
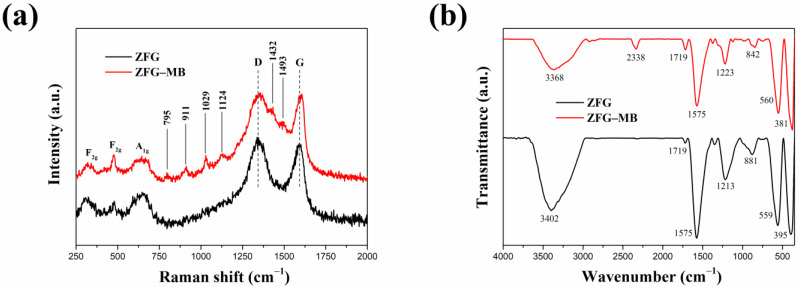
(**a**) Raman and (**b**) FT-IR spectra of ZFG and ZFG-MB.

**Figure 11:**
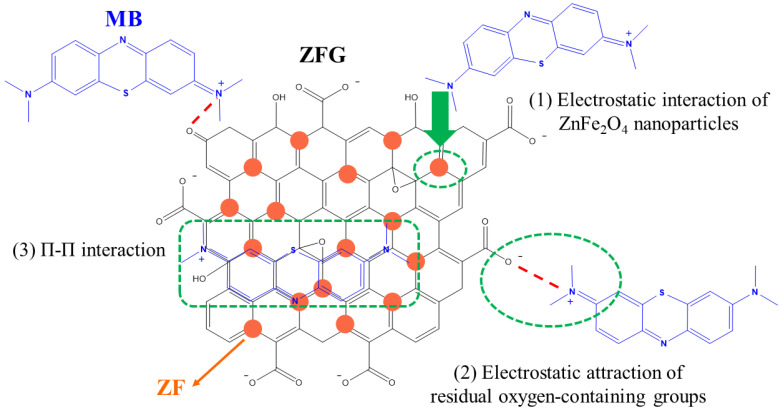
Proposed mechanism of MB dye adsorption on ZFG.

Generally, the dye adsorption mechanism of graphene-based compounds followed electrostatic interaction, π–π conjugation interactions, and hydrogen bonding [26]. Based on the adsorption of dyes by ZFG at the various initial pH (Figure 9a), it can be observed that the electrostatic force between the adsorbent and dye molecules plays a major role in the adsorption mechanism. The Raman spectrum can confirm the π–π interaction mechanism. According to Xiao et al., when the adsorption process had π–π interaction, the electronic structure of rGO could be changed such that the G peak of the adsorbent had shifted after adsorption [17]. This phenomenon is due to the electron coupling between MB C=C double bonds and benzene rings π electrons of rGO. Furthermore, when the graphene is an electron-donor in the adsorption process, the G band can shift to the lower frequency, while the electron-acceptors cause the G band to have a blue shift. Figure 10a shows the Raman spectra of ZFG before and after the adsorption process. The D band and the G band of ZFG are located at 1348 and 1589 cm^−1^. After adsorption, the G band of the nano-adsorbent shifts to 1600 cm^−1^. This result shows that the ZFG absorption mechanism has π–π interaction. In addition, the blue shift of the G band indicates that rGO behaves like an electron-acceptor in the MB adsorption process. The Raman spectra of MB powder are shown in Figure S4. There are six new peaks of ZFG after adsorption, located at 795, 911, 1029, 1124, 1432, and 1493 cm^−1^, which are from adsorbed MB. These peaks express the different vibration models of methylene blue, including stretching, twisting, and in-plane banding of C-H bond, carbon skeleton in-plane banding and stretching, C-S stretching, and N-C-H in-plane banding [111]. The appearance of these new peaks indicates the adsorption of MB molecules on the ZFG surface. In addition, the FT-IR spectra of Figure 10b shows the change of the adsorbent after adsorption of the dye and also indicates the electrostatic interaction. The intensity of the –OH group vibration peak, which is located about 3400 cm^−1^, has obviously decreased [112]. The adsorption and interaction mechanism of MB dye with ZFG can be deduced from the adsorption experiments and the results of Raman and FT-IR spectra, as shown in Figure 11. The first part of the mechanism is electrostatic interactions from the ZF nanoparticle and cationic MB [113]. The second part is the interaction between the residual oxygen-containing groups (–COOH and –OH groups) and MB = N + H group. The reaction formula between residual oxygen-containing groups and MB is shown below [113].

rGO–OH + MB^+^→rGO–O^−^ –MB^+^ + H^+,^ (12)

rGO–COOH + MB^+^→rGO–COO^−^ –MB^+^ + H^+.^ (13)

The third part is the electron donor-acceptor interactions (π–π conjugation) with rGO surfaces.

## 4. Conclusions

In this work, we used a simple method to prepare ZFG nanocomposites. All the various ZFG materials were successfully prepared using the microwave hydrothermal method from the results of XRD, Raman, and FT-IR. These nano-adsorbents showed a higher dye removal ability in removing MB. The adsorption rate and maximum adsorption capacity of ZFG-18 was 1.87 × 10^−1^ g mg^−1^ min^−1^ and 212.77 mg g^−1^, which is higher than other ZFGs as per previous reports. TEM results show that ZFG-18 has the smallest particles and a more homogeneous ZF distribution. Furthermore, the two main mechanisms of the MB adsorption process were observed by FT-IR, Raman spectroscopy, and the adsorption measurements at various pH values. The first mechanism is the electrostatic interaction between the MB dye = N + H group and the residual oxygen functional group of rGO. The second mechanism is the π–π interactions between the rGO carbon skeleton and dye molecules. The VSM measurements and recovery tests have shown that ZFG can be separated by magnets and has a long recovery life. Therefore, ZFG has the potential to be used in wastewater treatment.

## Figures and Tables

**Table 1:** D and G band location and I_D_/I_G_ value of GO, rGO, and ZFGs.

Sample	D Band (cm^−1^)	G Band (cm^−1^)	I_D_/I_G_
GO	1361	1605	0.99
rGO	1346	1577	1.03
ZFG-14	1346	1587	1.03
ZFG-16	1343	1588	1.03
ZFG-18	1348	1589	1.04
ZFG-20	1344	1588	1.05

**Table 2:** Average particle size of ZF, ZFG-14, ZFG-16, ZFG-18, and ZFG-20.

Sample	Particle Size (nm)
ZF	12.1 ± 2.7
ZFG-14	10.6 ± 2.0
ZFG-16	10.0 ± 1.9
ZFG-18	8.1 ± 1.8
ZFG-20	9.3 ± 1.8

**Table 3:** Adsorption capacity and removal percentage of as-prepared samples.

Sample	q_e_ (mg g^−1^)	Removal (%)
rGO	43.06 ± 0.13	86.12 ± 0.27
ZF	9.49 ± 0.79	18.98 ± 1.57
ZFG-14	47.21 ± 0.04	96.31 ± 0.07
ZFG-16	48.32 ± 0.01	98.57 ± 0.03
ZFG-18	49.06 ± 0.02	98.13 ± 0.04
ZFG-20	48.63 ± 0.02	98.24 ± 0.03

**Table 4:** Adsorption kinetic parameters of rGO, ZF, and ZFGs.

Models	Parameters	Sample					
		rGO	ZF	ZFG-14	ZFG-16	ZFG-18	ZFG-20
Pseudo-first-order	*q_e_* (mg g^−1^)	53.70	4.87	62.88	29.40	5.03	3.73
	*k*_1_ (min^−1^)	1.13 × 10^−1^	1.04 × 10^−1^	1.83 × 10^−1^	2.28 × 10^−1^	1.58 × 10^−1^	1.28 × 10^−1^
	R^2^	0.9505	0.7489	0.8743	0.9603	0.5975	0.6833
Pseudo-second-order	*q_e_* (mg g^−1^)	69.93	10.65	55.24	51.02	49.26	49.26
	*k*_2_ (g mg^−1^ min^−1^)	2.15 × 10^−3^	4.93 × 10^−2^	3.62 × 10^−3^	1.47 × 10^−2^	1.87 × 10^−1^	6.24 × 10^−2^
	R^2^	0.9909	0.9916	0.9980	0.9987	0.9999	0.9998
Elovich	*α* (g mg^−1^ min^−1^)	8.31	3.86 × 10^4^	28.99	357.46	1.96 × 10^16^	2.47 × 10^7^
	*β* (g mg^−1^)	6.68 × 10^−2^	1.46	9.06 × 10^−2^	1.45 × 10^−1^	0.8195	3.92 × 10^−1^
	R^2^	0.9790	0.5788	0.9972	0.8112	0.6572	0.7152

**Table 5:** Fitting parameters of Langmuir, Freundlich, and Temkin adsorption isotherm of as-prepared samples.

Models	Parameters	Sample					
		rGO	ZF	ZFG-14	ZFG-16	ZFG-18	ZFG-20
Langmuir	*q_m_* (mg g^−1^)	120.48	3.49	113.64	98.04	212.77	208.33
	*K_L_* (L mg^−1^)	2.83 × 10^−1^	1.40	8.71 × 10^−1^	7.45 × 10^−1^	2.61	7.62 × 10^−1^
	*R_L_*	5.56 × 10^−2^ ~ 2.61 × 10^−1^	1.25 × 10^−1^ ~ 4.16 × 10^−1^	1.88 × 10^−2^ ~ 1.03 × 10^−1^	2.19 × 10^−2^ ~ 1.18 × 10^−1^	6.30 × 10^−3^ ~ 3.69 × 10^−2^	2.14 × 10^−2^ ~ 1.16 × 10^−1^
	R^2^	0.9971	0.9930	0.9824	0.9652	0.9999	0.9977
Freundlich	n (mg^−1^)	2.89	3.42	5.32	3.57	8.34	2.27
	*K_F_* (mg g^−1^)	36.14	2.00	65.95	46.43	154.93	54.51
	R^2^	0.8926	0.9903	0.2071	0.3469	0.9241	0.6586
Temkin	*A_T_* (L g^−1^)	3.36	20.52	1091.80	25.32	1662.03	0.96
	*B* (kJ mol^−1^)	24.22	6.74 × 10^−1^	11.12	16.56	21.02	67.87
	R^2^	0.9549	0.9825	0.1089	0.2122	0.9411	0.6532

**Table 6:** Maximum adsorption capacity of MB on various adsorbents.

Adsorbents	Adsorption Capacity, q_max_ (mg g^−1^)	References
ZFG-18	212.77	This work
Activated carbon	2.57	[13]
AC-MnFe_2_O_4_	77.44	[14]
Zerolit DMF exchange resin	24.75	[18]
CoFe_2_O_4_/GO	156.74	[27]
GO-Fe3O4@P4VP	164.20	[29]
rGO-Fe_2_O_3_-Fe_3_O_4_	72.8	[31]
ZnO/ZnFe_2_O_4_	37.272	[101]
ZnAl/DS LDH	113	[102]
Epichlorohydrin crosslinked chitosan/carbon–clay	86.08	[103]
Graphene	153.85	[104]
Modified lychee seeds	124.5	[105]
Activated Carbon-clay	178.65	[106]
Fe_3_O_4_/kaolinite nanocomposite	42.3	[107]
TiO_2_-PVA	138.888	[108]
Gl-crosslinked PVA/VC-MWCNTs composite	16.844	[109]

## Data Availability

All the data are available within the manuscript.

## Supplementary Materials

The following are available online at https://www.mdpi.com/article/10.3390/ma14185394/s1, **Figure S1**: The Raman spectrum of ZF and ZFGs at 250~1000 cm^−1^. Figure S2: TEM image of GO. Figure S3: The particle distribution of (**a**) ZF, (**b**) ZFG-14, (**c**) ZFG-16, (**d**) ZFG-18, and (**e**) ZFG-20. Figure S4: The Raman spectra of solid MB powder.
