# Supplementary material for: Facile Microwave Hydrothermal Synthesis of ZnFe2O4/rGO Nanocomposites and Their Ultra-Fast Adsorption of Methylene Blue Dye
Source: Materials (Basel). 2021 Sep 18;14(18):5394. doi: 10.3390/ma14185394 (PMC8467475; doi:10.3390/ma14185394)
Supplement: Supplementary file 1 [file materials-14-05394-s001.zip › materials-1343496-supplementary.pdf]

Supplementary

Facile Microwave Hydrothermal Synthesis of $ZnFe_2O_4$/rGO Nanocomposites and Their Ultra-Fast Adsorption of Methylene Blue Dye

En-Rui Wang and Kun-Yauh Shih *

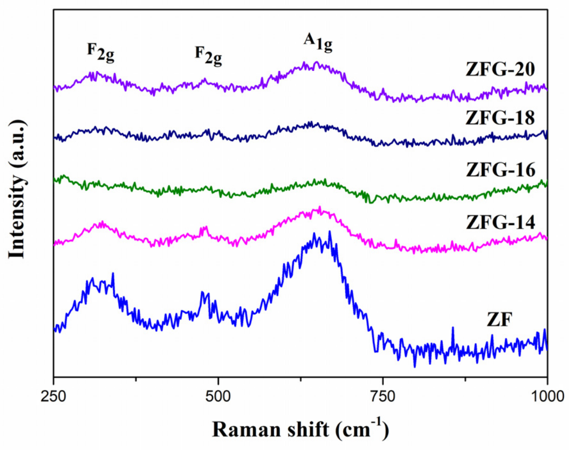

Figure S1. The Raman spectrum of ZF and ZFGs at 250~1000 cm^{-1}.

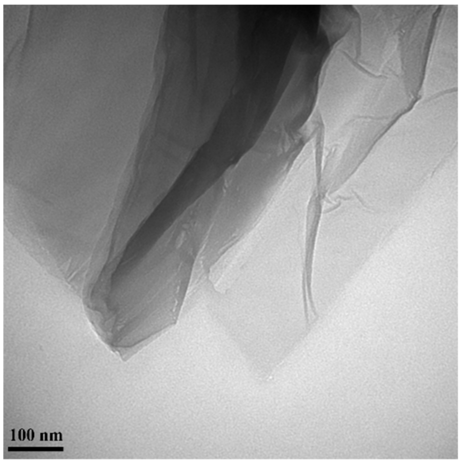

Figure S2. TEM image of GO.

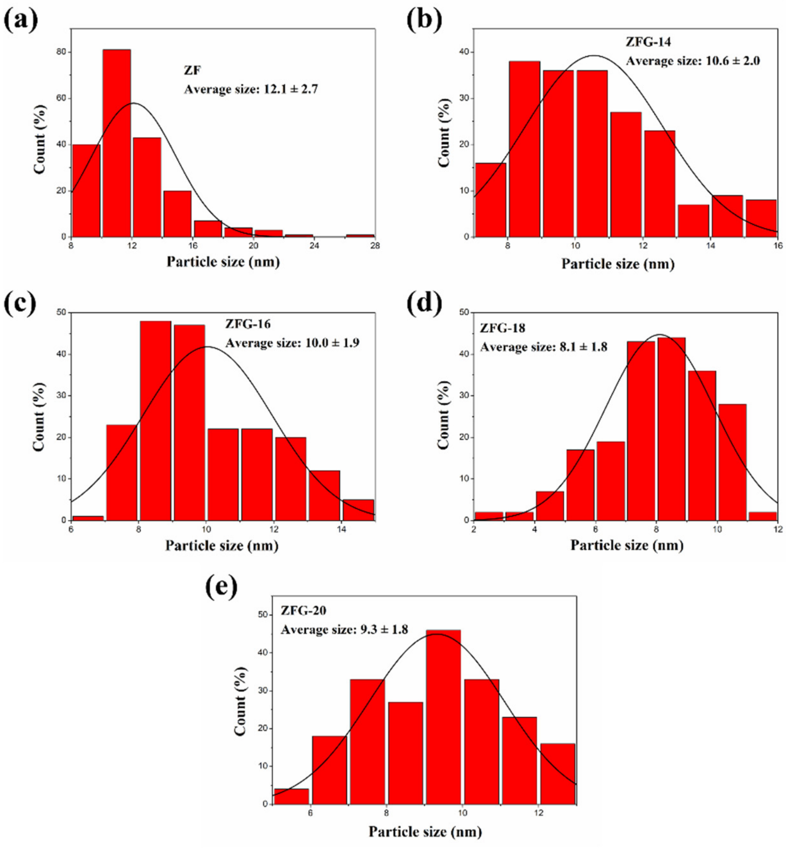

Figure S3. The particle distribution of (**a**) ZF, (**b**) ZFG-14, (**c**) ZFG-16, (**d**) ZFG-18, and (**e**) ZFG-20.

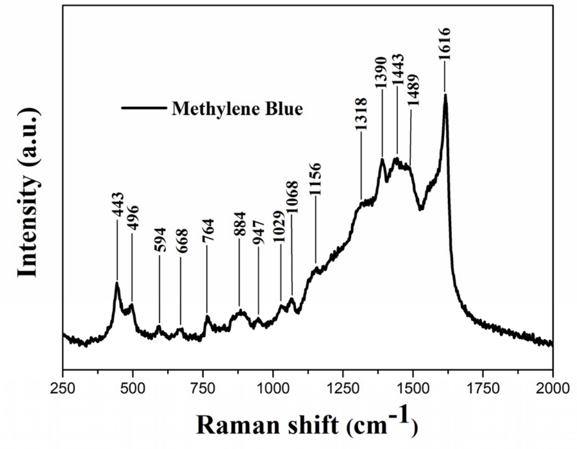

Figure S4. The Raman spectra of solid MB powder.
